# Outcomes of epiretinal proliferation embedding technique in the surgery for full-thickness macular hole

**DOI:** 10.1038/s41598-024-58449-1

**Published:** 2024-04-08

**Authors:** Jaehwan Choi, Sang Jin Kim, Se Woong Kang, Sungsoon Hwang, Ki Young Son

**Affiliations:** 1 Department of Ophthalmology, Kyung Hee University Medical Center, Kyung Hee University, Seoul, Korea; 2 Department of Clinical Research Design and Evaluation, SAIHST, Sungkyunkwan University, Seoul, Korea; 3 Department of Ophthalmology, Samsung Medical Center, Sungkyunkwan University School of Medicine, Seoul, Korea; 4 Department of Ophthalmology, Chungnam National University Sejong Hospital, Sejong, Korea

**Keywords:** Outcomes research, Retinal diseases

## Abstract

To compare visual and anatomical outcomes between peeling and embedding of epiretinal proliferation in patients with full-thickness macular holes (FTMH) with epiretinal proliferation (EP), this retrospective cohort study classified patients into two groups based on whether EP was completely peeled (peeling group, n = 25 eyes), or embedded into the hole (embedding group, n = 31 eyes) during surgery. Preoperative characteristics and postoperative outcomes, including best-corrected visual acuity and the length of the disrupted external limiting membrane and ellipsoid zone, were compared. Preoperative features including visual acuity and hole size did not differ between the two groups. All studied eyes achieved closure of the macular hole postoperatively. Visual acuity significantly improved at 3, 6, and 12 months postoperatively in both groups. The visual acuity 1-month after surgery was better in the embedding group than that in the peeling group (0.28 ± 0.29 vs. 0.50 ± 0.42 logarithm of the minimum angle of resolution, *P* = 0.016), although the difference was not noted after 3 months postoperatively. The embedding group showed shorter disruption of the external limiting membrane than the peeling group postoperatively (62.6 ± 40.2 μm vs. 326.2 ± 463.9 μm at postoperative 12 months, *P* = 0.045). In conclusion, the embedding technique during surgical repair of a FTMH with EP facilitates recovery of the outer foveal layers and promotes earlier restoration of visual function.

## Introduction

Epiretinal proliferation (EP) is located at the edge of the lamellar macular hole (LMH) or full-thickness macular hole (FTMH) and manifests as a homogeneous, medium reflective material on top of the internal limiting membrane on Fourier-domain optical coherence tomography (OCT)^1–5^. It is also known as atypical epithelial tissue^6–8^. Previous histopathologic studies on EP have revealed that it is mainly composed of migrated Müller cells from the retina^9,10^. In addition, OCT studies have shown that FTMH with EP frequently accompanied complete posterior vitreous attachment or detachment^11^. The developmental mechanism of FTMH with EP is different from that of idiopathic FTMH, which is mainly caused by vitreofoveal traction^12,13^. To date, FTMH with EP is regarded as a manifestation of slowly progressive foveal degeneration that evolves from LMH with EP^11,14–16^.

Previous studies have shown that FTMH with EP had worse surgical outcomes than those without EP, including worse postoperative visual acuity and larger disruption of the external limiting membrane (ELM), ellipsoid, and interdigitation zones^12,17^. In contrast to FTMH without EP, which develops rather abruptly by vitreofoveal traction^13^, FTMH with EP are caused by relatively chronic and degenerative nature of disease progression and seems to be associated with its worse surgical outcomes^11^.

The main cellular constituent of EP originates from Müller cells^9^, an indispensable cellular component that maintains retinal architecture. This gave rise to the idea that embedding of EP in the hole during surgical repair would “return back” the escaped tissue^18^. The embedding technique on LMH with EP has been reported^19,20^. However, its benefit over total removal of EP in eyes with FTMH has not been studied. In this study, we compared the clinical outcomes of embedding and complete peeling of EP during surgical repair of FTMH with EP.

## Methods

### Setting

This was a retrospective cohort study of patients with FTMH with EP who underwent pars-plana vitrectomy and were followed up for at least 3 months after surgery. The study adhered to the tenets of the Declaration of Helsinki and was approved by the Institutional Review Board of Samsung Medical Center, Seoul, South Korea (IRB number 2022-11-069). The Institutional Review Board of Samsung Medical Center waived the need for informed consent owing to the retrospective design of this study.

### Participants

The electronic medical records of patients who underwent surgical repair for FTMH with EP between December 2014 and 2021 at Samsung Medical Center were retrospectively reviewed. The presence of EP was defined when well-demarcated homogenous medium reflective material was substantially located on the epiretinal surface around the hole^12^ in the preoperative B-mode or en-face optical coherence tomography (OCT). Patients were classified into the peeling group (the whole extent of EP was peeled off) or the embedding group (the EP was peeled centripetally up to the hole margin and trimmed to an adequate size. Subsequently, the trimmed residual epiretinal tissue was inverted and embedded into the hole). To exclude myopic FTMH, eyes with axial length > 26.5 mm were excluded. Patients who had undergone FTMH repair surgery before and had a history of retinal detachment, advanced glaucoma, age-related macular degeneration more severe than intermediate stage, and diabetic retinopathy equal or severe than severe non-proliferative diabetic retinopathy were excluded. Patients with secondary FTMH, resulting from trauma, solar retinopathy, and idiopathic juxtafoveal telangiectasia, were excluded. Patients who were followed up for less than 3 months after surgery were also excluded.

### Surgical technique

**Figure 1:**
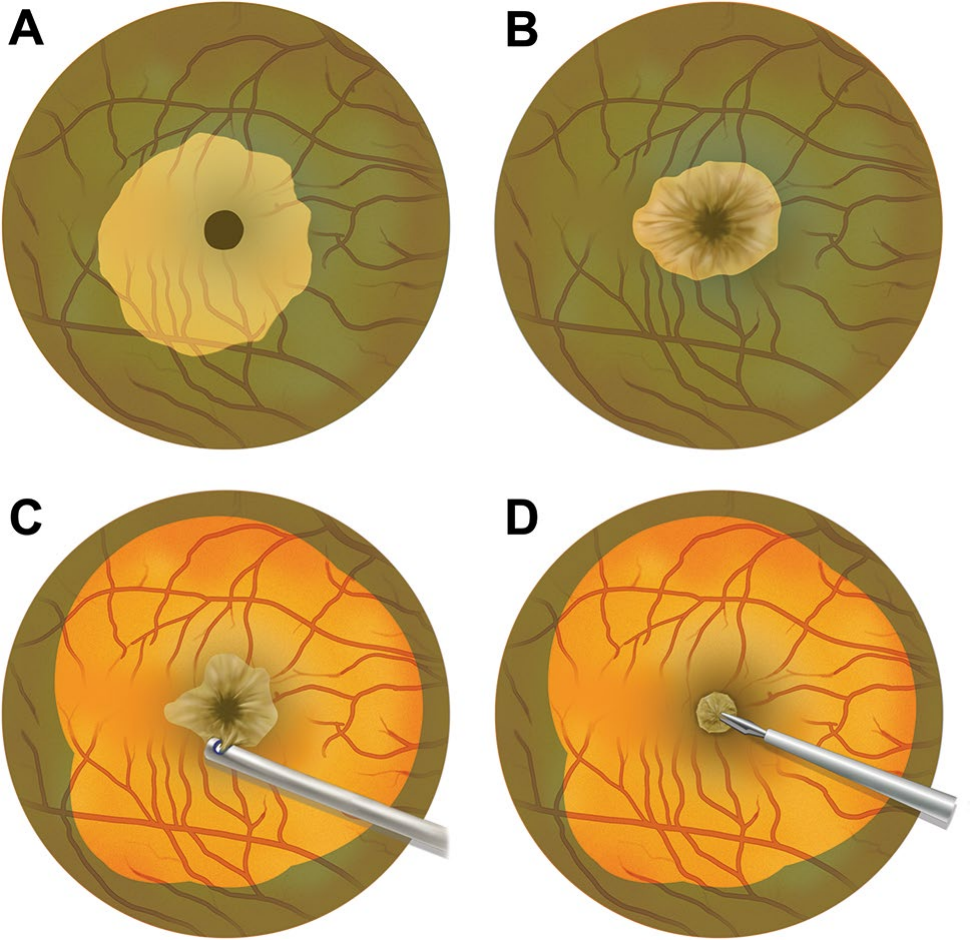
Epiretinal proliferation embedding in surgery for macular hole with epiretinal proliferation. (**A**) After initial staining with indocyanine green dye, unstained epiretinal proliferation (represented by the yellow membrane) is differentiated from the stained internal limiting membrane (represented by the green membrane). (**B**) The epiretinal proliferation is lifted and peeled from the internal limiting membrane up to the hole margin, and then the second indocyanine green dye staining was performed to stain the unstained portion of internal limiting membrane that was beneath the epiretinal proliferation during the first stain. (**C**) After completion of curvilinear internal limiting membrane peeling, the lifted epiretinal proliferation was trimmed using a vitreous cutter (**D**) The trimmed epiretinal proliferation is inverted and embedded into the macular hole.

Phacoemulsification and posterior chamber intraocular lens implantation were performed if the patient had vision-affecting cataracts or if the age of patients was older than 60 years. Surgery was performed by one surgeon (S.W.K). The procedure consisted of 23- or 25-gauge sutureless vitrectomy (Constellation Vision System; Alcon Laboratories, Inc., Fort Worth, TX, USA), internal limiting membrane peeling with the assistance of 0.3 mg/mL indocyanine green dye staining and intraocular gas tamponade (air, sulfur hexafluoride, or perfluoropropane gas). In eyes without complete posterior vitreous detachment (PVD), PVD induction and partial posterior hyaloidectomy were performed^21^. In cases where epiretinal membrane was present, it was completely removed. Discriminating between EP and epiretinal membrane was straightforward as epiretinal membrane was located outside the extent of EP. Additionally, EP appeared softer and thicker and exhibited a yellow color attributed to xanthophyll pigmentation^11^. EP was completely peeled in all eyes that underwent surgery from 2014 to 2018 (peeling group) and embedded in all eyes (embedding group) from 2019. There was no substantial difference between the two groups regarding any aspects of surgical technique and postoperative care, except for peeling or embedding of EP. Figure 1 illustrates the embedding technique in current study. Double staining of indocyanine green was employed to securely isolate EP and to completely peel off the parafoveal internal limiting membrane. Staining was first performed to distinguish the EP from the internal limiting membrane. Then, the margin of EP was lifted and peeled centripetally from the internal limiting membrane. The lifted EP and internal limiting membrane were completely peeled off and removed in the peeling group. When peeling EP attached to the margin of the macular hole, force was not solely applied in one direction. Instead, it was delicately applied from various direction around the hole to minimize foveal damage caused by retinal traction. EP peeling was carried out utilizing Maxgrip® forceps (Alcon Laboratories, Inc., Fort Worth, TX, USA). In the embedding group, we had the lifted EP tissue attached to the hole margin, Indocyanine green dye was then used again to stain the unstained internal limiting membrane that was beneath the EP during the first staining. After the second indocyanine green dye staining, the curvilinear peeling of internal limiting membrane was completed. Utilizing a vitreous cutter (Ultravit® Vitrectomy Probe; Alcon Laboratories, Inc., Fort Worth, TX, USA), the lifted EP tissue was trimmed so as to be accommodated into the hole. Then the inverted flap of EP tissue was embedded into the base of the hole, taking extra caution to avoid compression of the retinal pigment epithelium at the hole bed. Fluid–air and air–gas exchange was performed gently to avoid dislocating the embedded EP. All patients were instructed to maintain a prone position for 24 h post-surgery, followed by either prone or seated with facedown positioning for the subsequent 7 days, irrespective of tamponade type.

### Examinations

Preoperative demographic data, best-corrected visual acuity (BCVA), slit-lamp biomicroscopic examination, axial length, FTMH size, and manifest refraction were assessed. The axial length was measured using swept-source OCT (Argos; Movu, Inc., CA, U.S.A). B-mode macular images using spectral-domain OCT (Spectralis™; Heidelberg Engineering GmbH, Heidelberg, Germany) of 8 mm horizontal and vertical images crossing the central fovea and nineteen 6 mm horizontal raster images encompassing the macular area were taken in all patients. En-face OCT images were obtained using either spectral-domain OCT with 20° × 15° macular volume scans or swept-source OCT (DRI OCT Triton; Topcon Corporation, Tokyo, Japan) in the 6 mm × 6 mm macular region. FTMH size in this study was defined as the mean of the minimal hole diameters in the horizontal and vertical sections of B-mode OCT images.

Patients were followed up at 1 week and 1, 3, 6, and 12 months postoperatively. BCVA was measured at each postoperative visit, and OCT images were taken at all follow-up visits except at the week 1 visit. The ELM and ellipsoid zone disruption were measured as the mean length of disruption of each layer from horizontal and vertical OCT scans crossing the foveal center. Preoperative and postoperative OCT parameters were measured manually by two observers (S.H and K.Y.S) using built-in OCT software, and the mean value was used.

### Statistical analyses

Preoperative and postoperative data at 1, 3, 6, and 12 months were compared between the peeling group and the embedding group. BCVA was converted to the logarithm of the minimum angle of resolution (logMAR) scale for statistical analysis. Wilcoxon rank-sum test was used to compare the continuous variables between the two groups. The chi-square test was used to compare categorical variables. Wilcoxon signed-rank test was used to compare postoperative BCVA with the preoperative BCVA in each group. Statistical analyses were performed with R software (version 4.2.1; R Core Team (2022). R: A language and environment for statistical computing. R Foundation for Statistical Computing, Vienna, Austria. URL https://www.R-project.org/). Statistical significance was set at *P* value less than 0.05.

### Ethics declarations

This research followed the tenets of the Declaration of Helsinki and was approved by Institutional Review Board (IRB) of the Samsung Medical Center, Seoul, South Korea Informed consent was waived by the board as the research was retrospective, anonymous, and presented no threat to the rights and welfare of the research participants.

## Results

Fifty-six eyes of 56 patients met the inclusion criteria. The mean age was 64.0 ± 9.4 years, and 29 (51.8%) patients were female. Of 56, 31 (55.4%) patients were in the embedding group and 25 (44.6%) were in the peeling group. Table 1 shows the baseline demographic and clinical characteristics of the two groups. The mean age and sex ratio did not different between the two groups. The mean preoperative BCVA was 0.41 ± 0.30 logMAR (20/50) in the embedding group and 0.47 ± 0.28 logMAR (20/63) in the peeling group (*P* = 0.339). The mean hole size and axial length did not differ between the two groups.

**Table 1:** Baseline characteristics of the embedding and peeling groups.

	Embedding group	Peeling group	*P* value
Eyes, n	31	25	
Age, years (SD)	62.6 (9.9)	65.8 (8.6)	0.194
M:F, n	15:16	12:13	1.000
BCVA			0.339
logMAR (SD)	0.41 (0.30)	0.47 (0.28)	
Snellen (range)	20/50 (20/320–20/20)	20/63 (20/200–20/20)	
Hole size, μm (SD)	254.1 (192.7)	260.3 (187.7)	0.904
Axial length, mm (SD)	24.3 (1.3)	23.8 (0.8)	0.122
Lens status			0.945
Phakia	20 (64.5%)	15 (60%)	
Pseudophakia	11 (35.5%)	10 (40%)	
Gas tamponade, n			0.563
SF_6_	22 (71.0%)	15 (60.0%)	
C_3_F_8_	9 (29.0%)	10 (40.0%)	

*n* number, *y* year, *M* male, *F* female, *BCVA* best corrected visual acuity, *logMAR* logarithm of the minimum angle of resolution, *SF*_*6*_ sulfur hexafluoride, *C*_*3*_*F*_*8*_ perfluoropropane, *SD* standard deviation.

**Figure 2:**
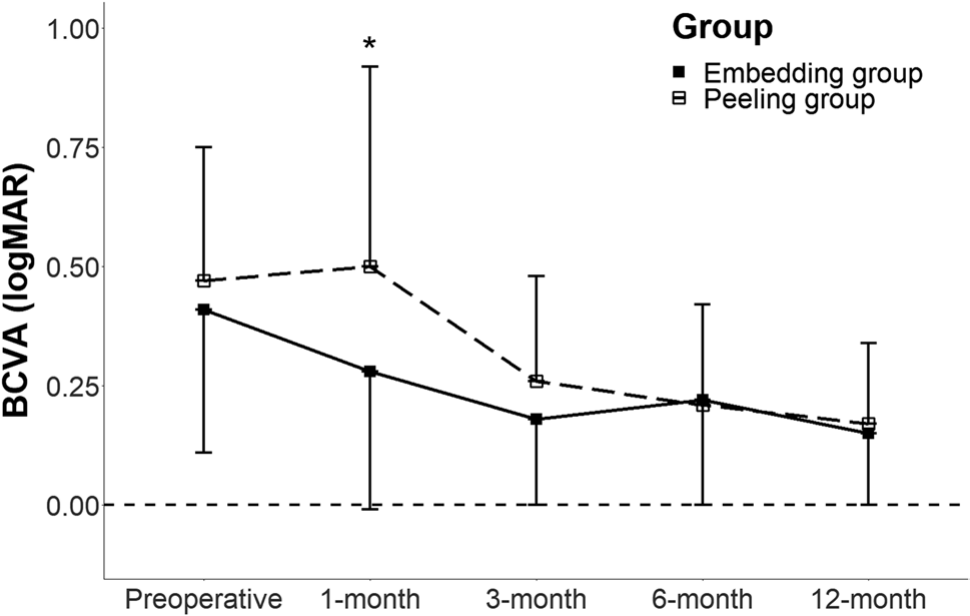
Comparison of pre- and postoperative BCVA between the embedding and peeling groups. After surgical repair, BCVA was significantly improved in both groups at 3, 6, and 12 months. However, after 1 month postoperation, only the embedding group showed improved BCVA and it was significantly better than that of the peeling group. *BCVA* best-corrected visual acuity, *logMAR* logarithm of the minimal angle of resolution, *n* number. *Statistically significant at *P* < 0.05.

All 56 eyes achieved closure of FTMH after surgery. Compared with baseline, BCVA was significantly improved at 3, 6, and 12 months postoperatively in both groups. In the embedding group, preoperative BCVA was 0.41 ± 0.30 logMAR. After surgery, BCVAs at 1, 3, 6, and 12 months were 0.28 ± 0.29, 0.18 ± 0.17, 0.22 ± 0.22, and 0.15 ± 0.17 logMAR, respectively (P = 0.004, 0.001, 0.006, and 0.004, respectively). In contrast, the peeling group had a preoperative BCVA of 0.47 ± 0.28 logMAR. Postoperative BCVAs at 1, 3, 6, and 12 months were 0.50 ± 0.42, 0.26 ± 0.22, 0.21 ± 0.21, and 0.17 ± 0.17, respectively (P = 0.901, 0.001, 0.001, and 0.008, respectively). The mean postoperative BCVA 1 month after surgery was better in the embedding group than that in the peeling group (0.28 ± 0.29 logMAR (20/40) vs. 0.50 ± 0.42 logMAR (20/63), respectively, *P* = 0.016). Three months after surgery and afterwards, however, no difference was noted in the mean postoperative BCVAs between the two groups (Fig. 2). The visual change was also better in the embedding group than in the peeling group 1 month after surgery (− 0.11 ± 0.19 logMAR vs. 0.02 ± 0.28 logMAR, *P* = 0.037, respectively). However, there was no difference at 3, 6, and 12 months after surgery.

**Figure 3:**
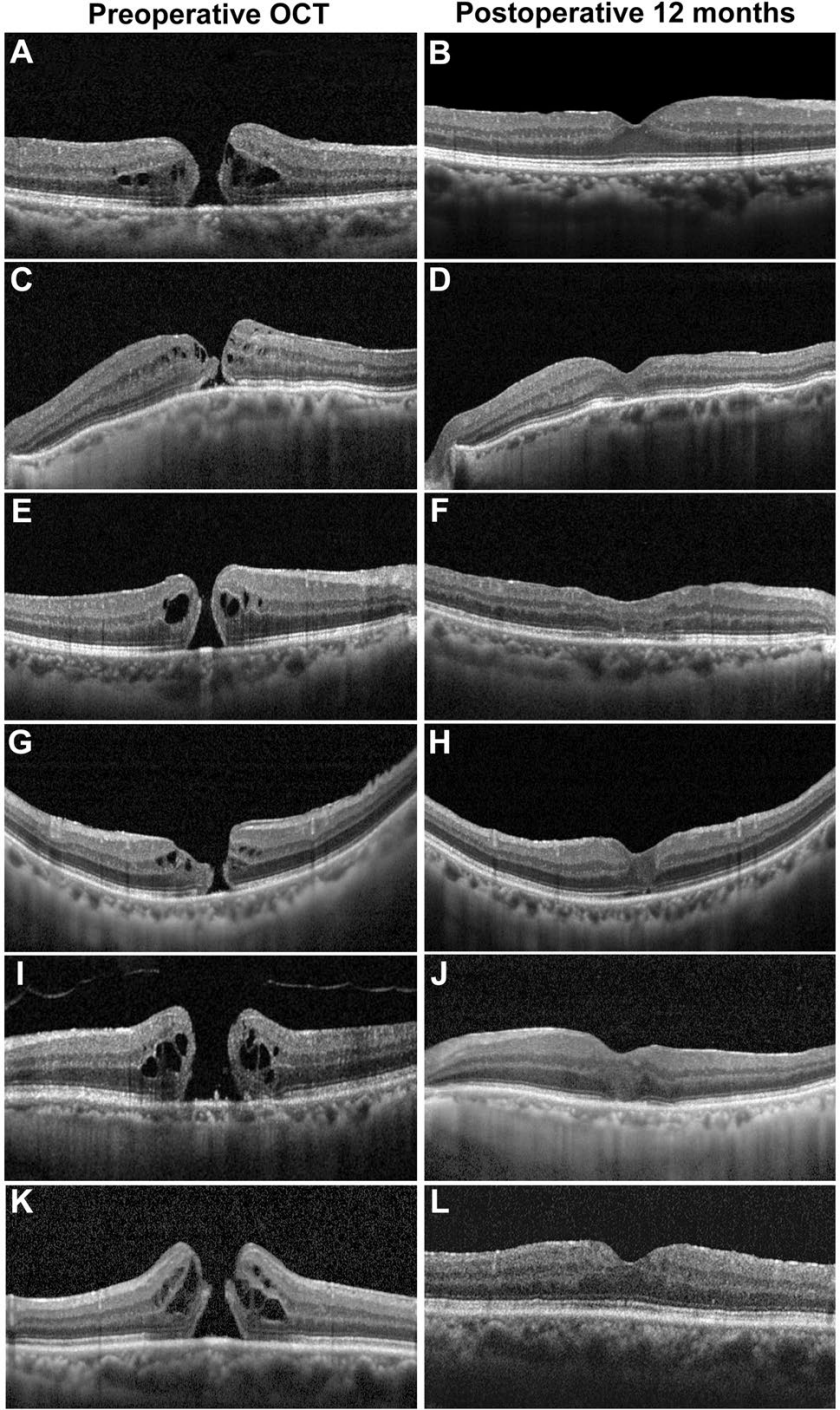
Representative cases from the embedding and peeling groups. (**A**–**F**) The embedding group. (**A**, **C**, **E**) Preoperative OCT images show full-thickness macular hole with epiretinal proliferation. (**B**, **D**, **F**) Postoperative 12-month OCT images show sealed macular hole, with defect at the external limiting membrane and ellipsoid zone in some cases (**D**, **F**). (**G**–**L**): The peeling group. (**G**, **I**, **K**) Preoperative OCT images. (**H**, **J**, **L**) Postoperative 12-month OCT images. The defects in the ellipsoid zone (**H**, **J**, **L**) and external limiting membrane (**H**, **J**) were presented. Note that the extent of ellipsoid zone is larger than that of the external limiting membrane (**H**, **J**). OCT, optical coherence tomography.

OCT examinations revealed that the mean ELM disruption length was significantly shorter in the embedding group at all postoperative follow-ups. The mean ellipsoid zone disruption length was shorter in the embedding group 1 month after surgery (465.7 ± 265.5 μm vs. 691.6 ± 424.8 μm, respectively, *P* = 0.045). However, this difference was not noted 3 months after surgery (Table 2). Figure 3 shows representative cases from the embedding group and the peeling group.

**Table 2:** Comparison of postoperative anatomical outcomes between the embedding and peeling groups.

	Embedding group	Peeling group	*P* value
Disruption of the ELM, μm (SD)			
1 month	316.9 (167.8)	533.0 (391.1)	0.027
3 months	177.8 (132.8)	418.3 (329.8)	0.002
6 months	127.9 (122.6)	280.0 (258.6)	0.017
12 months	62.6 (40.2)	326.2 (463.9)	0.045
Disruption of the ellipsoid zone, μm (SD)			
1 month	465.7 (265.5)	691.6 (424.8)	0.045
3 months	320.3 (200.7)	527.0 (409.7)	0.060
6 months	217.4 (145.5)	335.4 (342.1)	0.295
12 months	131.8 (88.0)	481.1 (519.2)	0.110

*ELM* external limiting membrane, *SD* standard deviation.

## Discussion

The gender distribution of the study population is nearly 1:1. This finding aligns with the results observed in previous research related to EP associate with FTMH^17^. Moreover, it starkly demonstrates a distinct difference from the FTMH without EP, which exhibits a clear female dominance^22,23^. Several studies have compared the visual prognosis of FTMH with EP and those without EP and showed that patients with FTMH with EP had poorer postoperative visual acuity than FTMH patients without EP^11,12^. In addition, the pathophysiology of FTMH with EP has been reported to be different from that of FTMH without EP^8,9^. Therefore, it may not be appropriate to apply the same surgical technique to FTMH with EP as those without EP.

In this study, both the embedding and peeling groups achieved complete FTMH sealing and improved visual acuity after surgery. However, the embedding group showed better BCVA improvement than the peeling group at postoperative 1 month. In other words, the embedding technique promoted faster visual recovery after surgery than complete peeling of epiretinal proliferation. It remains unclear how the embedding technique expedited visual recovery. One possible explanation is that the concept of the embedding technique that brings back the dislocated glial tissue to its original position could contribute to early vision recovery by promoting restoration of the outer retinal layers. Another hypothesis is that embedding technique aids in preventing foveal tractional damage, which may result from the peeling or EP.

Postoperative OCT examination revealed that the ELM disruption length was significantly shorter in the embedding group than in the peeling group at all follow-ups. Furthermore, the embedding group had a shorter ellipsoid zone disruption length than the peeling group, although statistical significance was noted only 1 month postoperatively. The ELM is composed of a connection between the photoreceptor inner layer and Müller cell process^24^. Previous studies on EP showed that an increase in its extent correlates with the worsening of foveal cavitation^8,14^. Consequently, EP is considered to consisted of centrifugally dislocated Müller cells rather than Müller cells proliferation. Repositioning of EP to its original position might help restore ELM. The ELM and ellipsoid zone integrity are well-known predictors of the visual prognosis in idiopathic FTMH and LMH^25–30^. This study supports that, as with idiopathic FTMH and LMH, postoperative visual outcome in FTMH with EP was also associated with the integrity of the ELM and ellipsoid zone. In particular, ellipsoid zone integrity was closely related to postoperative visual changes in patients with FTMH with EP. A previous study showed that intact ELM is indispensable for ellipsoid zone recovery after FTMH repair surgery^31^. Therefore, it can be assumed that the restoration of ELM precedes the ellipsoid zone restoration, and the EP embedding technique might expedite both ELM and ellipsoid zone recovery.

Double staining with indocyanine green can be a useful method to visualize both EP and the internal limiting membrane underneath the epiretinal proliferation. That is, the double staining with indocyanine green helps us to differentiate and isolate EP from internal limiting membrane by first stain, and then to visualize internal limiting membrane beneath the EP by second stain after lifting up EP^11^.

Because EP is connected to the inner retina at the margin of the FTMH^8^, there is a risk that the retinal tissue at the margin of the hole is lifted up by the tractional force when EP is peeled in the peeling group. Previous research demonstrated that internal limiting membrane peeling during surgery for lamellar macular holes can lead to the development of FTMH postoperatively^6,32^. Given the strong attachment of EP to inner retina at the macular hole margin^11^, caution is required to minimize tractional during EP peeling. Therefore, in the peeling group, efforts were made to reduce tractional foveal damage by carefully pulling EP attached to the macular hole margin from multiple directions rather than one direction. In this process, Maxgrip® forceps offered an advantage over end-gripping forceps due to its broader grasping extent, thereby aiding in prevention of EP tearing. On the other hand, in the embedding group, caution is required because of the potential risk of unintentional trauma to the hole base, especially the retinal pigment epithelium, during the embedding process. Although the embedding technique expedites earlier postoperative visual recovery than the peeling group, both peeling and embedding procedures may impose an inherent risk of surgical trauma. To figure this out, the use of intraoperative OCT would be helpful.

This study had several limitations. First, this was a retrospective study with a small number of participants. Second, the results were obtained over a relatively short follow-up period. All patients were followed up until 3 months postoperatively, and 44 (78.6%) and 27 (48.2%) patients were followed up at 6 and 12 months, respectively. This may have limited the power of our statements. Beyond the surgical technique, the impact of additional pre- and post-operative structural features which were not evaluated in the current study may influence surgical outcomes^17,33^. An additional prospective comparative study with a larger sample size, considering various pre- and post-operative anatomical features is needed.

Despite these limitations, this study is worthwhile as the first report demonstrating the safeness and usefulness of the embedding technique to promote better restoration of the outer retinal layers and earlier visual recovery in FTMH with EP. Because faster recovery of vision after FTMH surgery can improve patients’ quality of life and allow early return to work or daily life, the benefit of the embedding technique in patients with FTMH with EP is warranted.

In conclusion, current study suggests that the embedding technique used during the surgery for FTMH with EP may promote early visual restoration and anatomical recovery of outer retina.

## Data Availability

The datasets generated and/or analyzed during the current study are available from the corresponding author on reasonable request.
